# Computer-interpretable guidelines: electronic tools to enhance the utility of thyroid nodule clinical practice guidelines and risk stratification tools

**DOI:** 10.3389/fendo.2023.1228834

**Published:** 2023-08-15

**Authors:** Jeffrey R. Garber, Vivek Patkar

**Affiliations:** ^1^ Atrius Health, Beth Israel Deaconess Medical Center, Harvard Medical School, Boston, MA, United States; ^2^ Deontics, London, United Kingdom

**Keywords:** thyroid nodule, clinical practice guideline, clinical calculators, clinical decision support, computer interpretable guideline, evidence based care, guideline compliance, CDSS

## Abstract

Clinicians seeking guidance for evaluating and managing thyroid nodules currently have several resources. The principal ones are narrative clinical guidelines and clinical risk calculators. This paper will review the strengths and weaknesses of both. The paper will introduce a concept of computer interpretable guideline, a novel way of transforming narrative guidelines in to a clinical decision support tool that can provide patient specific recommendations at the point of care. The paper then describes an experience of developing an interactive web based computer interpretable guideline for thyroid nodule management, called Thyroid Nodule Management App (TNAPP). The advantages of this approach and the potential barriers for widespread adaptation are discussed.

## Background

Thyroid nodules are a common clinical problem. Increasing availability and the use of ultrasensitive imaging modalities have resulted in the over-detection of incidental thyroid nodules. A meta-analysis showed that 68.8% of all thyroid nodules undergoing surgical excision represented benign disease (1). Over diagnosis and over-treatment of thyroid nodules is a well-known challenge and has economic as well as individual health consequences (2). Deciding on the optimal management of a thyroid nodule and avoiding both unnecessary evaluation and treatment of benign nodules as well as missing thyroid cancer could be a challenging task for a nonspecialist. Clinicians seeking guidance for evaluating and managing thyroid nodules have a number of resources currently available to help them in their clinical decision-making. Available clinical resources fall into two broad formalisms: (A) Narrative clinical practice guidelines (CPGs)^1^ and (B) Clinical risk calculators ^2^. This paper provides a brief overview of both, highlighting the strengths and weaknesses of each formalism. The paper then introduces a lesser-known formalism known as computer-interpretable guideline (CIG), a derivative of conventional CPG, which harnesses the power of computational logic, workflow engines^3^, and artificial intelligence (AI) to deliver patient-specific recommendations at the point of care. CIGs can overcome many of the limitations of narrative guidelines and clinical calculators. Lastly, the paper discusses the advantages and the barriers to the adaptation of CIGs into clinical practice.

## Clinical practice guidelines

The CIGs are “systematically developed statements to assist practitioner and patient decision-making (3) and are usually published in the form of narrative documents. The CPGs provide a number of actionable recommendations of varying degrees of evidence strength, ranging from high-quality evidence (RCTs and meta-analysis) to expert opinions. CPGs are typically developed by a variety of specialist bodies such as professional associations, healthcare providers, or the national bodies entrusted with the task of overseeing clinical standards. A typical guideline-developing group is often multidisciplinary in nature, and the guideline-development process requires an exhaustive literature review, evaluation of evidence, and a consensus process. CPGs may include “clinical algorithms” in the form of flowcharts or a risk stratification model; however, these are usually intended for humans to read, internalize, and apply their recommendations when the appropriate situation arises. Once the guideline is written and published, it is disseminated using various paper and electronic dissemination routes. A systematic review specifically looking at CPGs on the management of thyroid nodules identified 10 guidelines published by different professional organizations, and the overall quality ranged from 3.0 to 6.25 on a seven-point AGREE-II scale (4). The study found that CPGs varied in methodological quality, and increased efforts are required to improve the quality of recommendations on the diagnosis and management of thyroid nodules and cancer.

The primary intention of a CPG is to reduce unjustified variation, standardize clinical practice, improve the quality of care, and decrease the cost of care. While the intention is laudable, the evidence suggests that the effort that goes into creating them may not be matched by the level of usage and adherence in practice (5–7). There are a number of reasons contributing to the underutilization of CPGs.

- **Dissemination barriers:** The target clinicians are often unaware of the availability of CPGs, and even when they are aware, it is difficult to access, read, and apply the relevant recommendations embedded within a lengthy guideline document.
- **Workflow integration barrier:** The inability to integrate a narrative CPG into the clinical workflow through an electronic medical record (EMR) means the usage is entirely dependent upon clinicians’ initiative to remember guideline recommendations and apply them to appropriate clinical scenarios. This is an unrealistic expectation in a busy clinical practice, especially in a generalist environment where the clinician is managing a diverse group of conditions.
- **Ambiguity:** The conventional narrative format of CPGs may introduce the inevitable ambiguity associated with language, and different readers could interpret the same recommendation differently. Consistent with this observation, Huang et al., in their systematic review, found the thyroid nodule guidelines’ score on the clarity of presentation varied widely from 39% to 82% (4).
- **Oversimplification:** Another drawback of narrative CPGs is the oversimplification of the clinical logic underpinning the recommendations. A typical CPG generally presents recommendations in the form of narrative statements, flowcharts, and tables, limiting its ability to embody complex clinical logic in order to preserve the legibility of the guideline. As a result, guideline recommendations often do not cover complex clinical scenarios.
- **Lack of validation:** Moreover, there are no standard mechanisms to reliably measure guideline usage and adherence in different situations across different populations and cultures.
- **Lack of mechanism for feedback and refinements:** The lack of workflow integration makes it difficult to collect any user feedback on the validity of guideline recommendations in real-world clinical practice, thus missing an important opportunity to close the loop and continue the refinement of CPG content.

## Clinical calculators for risk stratifications

Clinical calculators have become ubiquitous and are used by practitioners in a variety of clinical activities, such as calculating risks, scores, and probabilities, classifying patients into prognostic categories, and calculating derived data such as BMI. Fracture Risk Assessment Tool (FRAX) for calculating the risk of osteoporotic fracture in those with osteopenia (8) and the American College of Cardiology (ACC) Atherosclerotic Cardiovascular Disease (ASCVD) risk estimator (9) for predicting cardiovascular events in those without known ASCVID calculators are the ones most frequently utilized. At the time this manuscript was written, FRAX had been employed over 11 million times in the USA and over 40 million times across the globe. The ACC risk estimator, which employs standard, discrete data that are recorded in medical records, can be interfaced with electronic health records such as the EPIC medical record version that one of the author’s healthcare system uses. In addition to providing a risk estimation of cardiac events, it provides guidance about the use of aspirin, statins, and blood pressure goals. Similarly, a thyroid nodule calculator determines the risk of cancer to guide patient management decisions.

The most commonly employed thyroid nodule calculator is the American College of Radiology (ACR) TI-RADS (10). An updated artificial intelligence version of this tool, AI-TIRADS (11, 12), that uses a modified scoring system, has recently been developed. There are a number of other thyroid nodule risk calculators. A Korean calculator (13) uses ultrasound features for the evaluation of thyroid nodules with the AUS/FLUS Bethesda III cytology classification. The Brigham and Women’s Hospital tool is based on 20,000 cases (14). It provides estimates for populations of patients that are based on relatively objective, reproducible data. Given the limited amount of detailed information, specifically excluding high-risk characteristics, it is not well tailored for an individual patient at high risk for having thyroid cancer. In contradistinction, a calculator from Spain (15) calls for a substantial amount of specific information, including whether there is a history of autoimmune disease or a family history of thyroid cancer. The Cleveland Clinic calculator (16), which was among the earliest thyroid nodule risk calculators developed over a decade ago, serves as an example of the evolution of risk estimation. It uses vascularity, which is no longer used for risk stratification or as a “scoreable item.”

Calculators have several advantages in terms of computability and automation, workflow integration, decidability, proven validity, and usage data. They are easy to use, readily accessible, and require little time to employ. Hence, calculators can serve as a “ point- of-service “ tool. Additionally, they are suited to engaging patients by illustrating how data impact decision-making. For example, would the approach be different if the nodule were bigger, grew larger, or the patient was 10 years older?

However, there are many limitations to thyroid nodule calculators in terms of their overall applicability in the wider management decisions

- **Limited input variables:** It may limit data evaluation to the most reproducible and therefore limited number of items. The Brigham and Women’s calculator is an example of this (14). It may restrict data evaluation to thyroid ultrasound features alone, such as TI-RADS and AI TI-RADS (10–12). It may focus on a single FNA result, such as AUS/FLUS, as seen with the Korean version (13).
- **Exclusion of symptoms, signs, and patient preferences:** Nearly all thyroid nodule calculators omit features impacting clinical decision-making such as symptoms, physical examination findings such as a firm or fixed nodule, patient anxiety, or cosmetic concerns.
- **Lack of explainability and actionable advice:** Most clinical calculators are black boxes from the end users’ point of view. They do not explain the reasoning used by the algorithm to provide the output to the user. Thus, they do not serve as a tool for teaching clinicians. Some may provide risk statistics but not guidance (13, 14), while others may provide guidance but not statistics (10). They may not provide guidance about follow-up and simply provide a statistic about malignancy risk, leaving decision-making to the clinician using the calculator (12, 13).
- **Dissemination:** Stand-alone clinical calculators that are not integrated into the clinical workflow face the same dissemination barriers as CPGs, as many target users may not be aware of their existence.
  We anticipate that calculators will continue to evolve, play a role as a clinician aid, proliferate in number, and serve as a tool to assist clinicians in managing thyroid nodules or other conditions. However, for the better adoption of the calculators, they are required to be automated through integration into clinical workflow and should be a part of the broader digital ecosystem within an EMR (17).

## Computer -interpretable guideline: a formalism that enhances narrative guidelines

Clinical decision support system (CDSS) is a term used to describe a diverse group of computer technologies designed to assist clinical decision-making at the point of care. CDSSs have evolved over the last four decades, starting from simple rule-based expert systems to more advanced knowledge representation and workflow management systems (18). Advanced CDSS technologies have made it possible to encode and transform complex narrative guidelines, written primarily for human understanding, into an executable, automated CIG. Many different CIG formalisms have been developed in academia (19) to represent different aspects of clinical guidelines, such as recommendations, evidence, and workflow. The CIG format mitigates many of the limitations of a CPG described earlier. Formal semantics that underpins CIG enforces disambiguation of the clinical guideline logic. For example, a guideline may call for obtaining a serum TSH value and performing an ultrasound. However, it may not specify whether the recommendation is to do so simultaneously or sequentially. While a CIG will clarify and automate the workflow and track recommendations. Also, when a CIG is deployed *via* CDSS and integrated in to an electronic medical record, it can automatically pull the investigation results and clinical data to generate relevant patient-specific recommendations and drive the clinical workflow. Studies have shown them to be effective in the management of chronic disease by improving adherence to CPGs (20, 21). CIGs may also facilitate the testing and validation of guideline recommendations, both prospectively and retrospectively, by comparing recommendation acceptance and outcome data. Potential benefits of CIG, in addition to being trackable, include their use as a stand-alone medical education tool, including, for example, instructing the trainees about the impact of varying data. They can be integrated into electronic health records, and their use may range from a single patient decision tool with no retained data to storing data on multiple patients. They ultimately hold promise to serve as a registry platform for the entire spectrum of practice sizes, large multispecialty delivery systems, regional and national databases, or research consortiums, thus ultimately becoming a key tool for studying the impact of algorithms and recommendations on clinical outcomes.

Typically, a narrative clinical practice guideline development process and the process of transforming the CPG into a computer-interpretable guideline are disconnected and sequential rather than a joint co-development process. A completed and published CPG is used as input to develop a CIG. Peleg et al., in their 2014 paper (22), describe their experience developing computer-interpretable guidelines based on already published narrative and evidence-based AACE, AME, and ETA guidelines for the diagnosis and management of thyroid nodules (23). One of the learning lessons from this exercise was that the narrative guideline development process may miss potential refinements and improvements of the guideline recommendations identified during the validation and vetting process of CIG development by the time the narrative guideline is finished, dusted, and already disseminated. The section below describes a novel approach to using retrospective data and the CIG toolset to define, validate, and refine the guideline recommendations.

## TNAPP: a novel experiment using CIG and CDS technology to vet and validate thyroid nodule diagnostic and management recommendations

The Thyroid Nodule App (TNAPP) (24) is a novel web-based, readily modifiable, interactive algorithmic tool developed to provide thyroid nodule recommendations using the PRO*forma* CIG formalism (25) and Deontics^®^ commercially available advanced AI-based CDDS technology . The Deontic CDS platform comes with an authoring toolset and a CDS execution engine. The authoring tool converts language or “human understandable guidelines “ to a “ computer-interpretable logical model.” A CDSS engine then runs the logical model using individual patient data to generate patient-specific recommendations. A goal-based cognitive argumentation framework (26) underpins the inference logic of the engine to come up with recommendations. An example of inference logic is illustrated by the following common-practice example. It is raining outside, and you want to stay dry. Variables are how hard it is raining, wind intensity, and the time that will be spent in the rain. The resources to keep you from getting wet are an umbrella, a raincoat that has a hood, and a rain hat. The “data” from the following two examples determine the programmed recommendations about what resources to employ. It is drizzling, and you will only be stepping outside to pick up your morning paper. You may opt out of using any resources or just a rain hat. There is a monsoon, and you are headed on foot to a destination one mile away. Parallelism would provide elected resources, e.g., all or just a raincoat and hood, or a raincoat with a hood and hat. The same approach can be applied to a narrative guideline. In the case of thyroid nodularity, there are clinical factors and ultrasound findings that influence the decision of whether to proceed with a biopsy. If it is done, what actions do the results call for? If a biopsy is not done, does the patient need any follow-up? What follow-up is recommended, and when should it happen?

**Figure 1:**
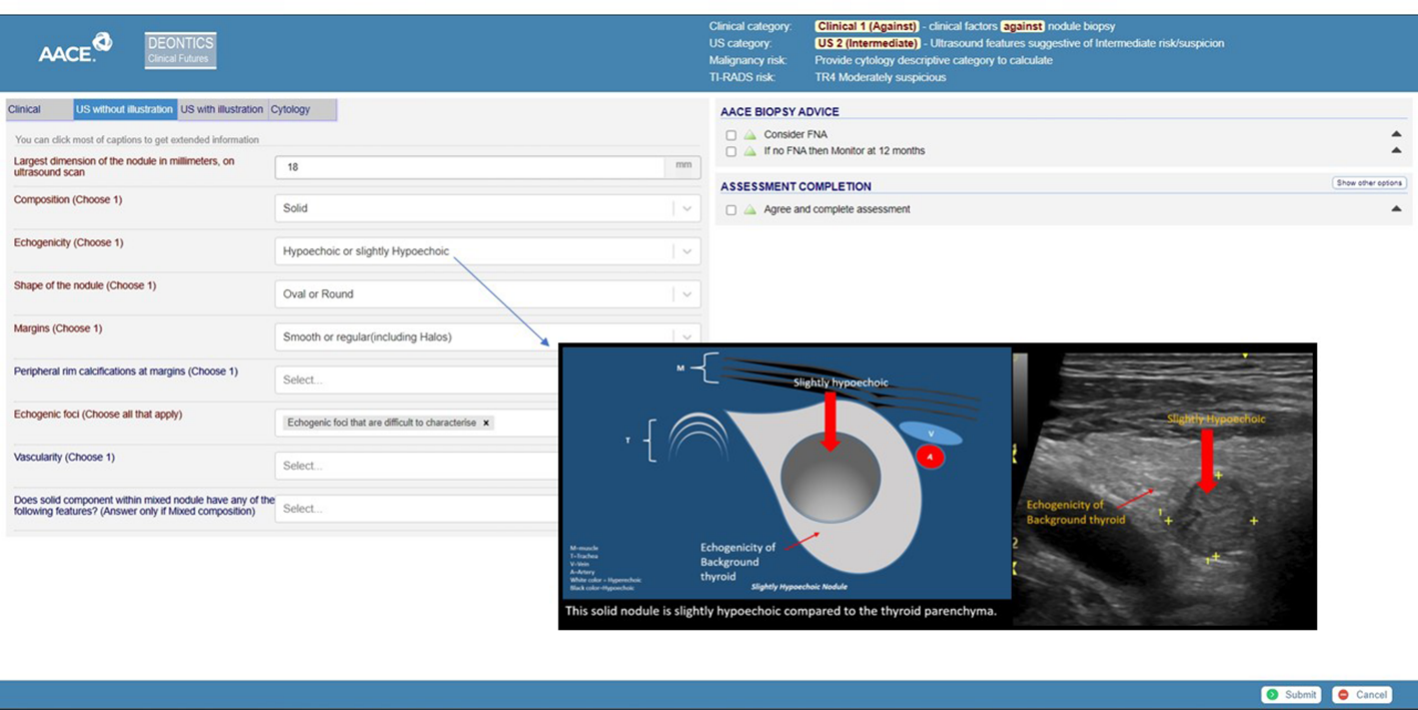
Screen capture of a demonstrator tool. The header shows various risk stratification calculator results: clinical and ultrasound risk stratification as clinical 1 and US2 (intermediate). The ACR-TIRADS risk is calculated as TR4. The left side of the screen shows the clinical, ultrasound (US), and cytology data capture tabs. The right side of the screen shows biopsy advice. The link to interactive TNAPP is https://aace-thyroid.deontics.com/.

A prototype CIG in the early stages of being vetted to evaluate a patient with a thyroid nodule (27, 28) provides a comprehensive approach for patients who meet inclusion criteria for employing the tool for decision-making. The variables are clinical factors supporting or not supporting performing a biopsy; ultrasound finding categorization as either low, intermediate, or high risk per AACE/AME guidelines; or a more stratified approach employing ACR TI-RADS. The initial recommendation is whether to perform a fine-needle aspiration (**Figure 1**). If not, the recommendations are whether follow-up is at all required and, if so, when it should be done. If an FNA is done, the cytologic Bethesda classification serves as the next determining variable for advice about follow-up and care.

The major challenge for employing CIG will be making it easy to use in a clinical setting. To be implemented in a time-constrained clinical setting, it must require minimal time to employ. It needs to be applicable in settings where resources are not identical. For example, do practitioners have access to or can patients afford molecular markers for evaluating indeterminate cytology? Are highly skilled surgeons available who can perform bilateral thyroidectomy with minimal morbidity when compared with surgery limited to unilateral lobectomy? More than one guideline can be used alone or alongside another, for example, AACE/AME or ACR TI-RADS can be used alone or together. Integration with electronic health records that provide substantial, if not complete, auto-population of requisite data, eliminating the time constraints clinicians face, will facilitate embracing and utilizing CIGs.

## Conclusion

CIG should become an adjunct rather than a replacement for clinical practice guideline development. They should be flexible tools that can be customized, readily accessed electronically, and easily modified as new guidance emerges. They have all these potential advantages in addition to facilitating expedited dissemination to a community of users whose feedback can accelerate their refinement, study outcomes, and influence how best to deliver cost-effective patient care when algorithmic approaches apply.

## Data availability statement

The original contributions presented in the study are included in the article/supplementary material. Further inquiries can be directed to the corresponding authors.

## Author contributions

Both authors have equally and substantially contributed to the concept, design and drafting of this paper and have approved the version to be published.

## Footnotes

1 CPG is a systematically developed narrative statement to assist clinicians and patients.

2 Clinical risk calculator is a computable model encoded in a software tool that takes discrete and nondiscrete data elements as its input and provides risk stratification and management advice as its output.

3 Workflow engine is a term commonly used to describe types of clinical decision support systems that can model and enact clinical processes or workflows at the point of care.
